# Expression of the platelet-activating factor receptor enhances benzyl isothiocyanate-induced apoptosis in murine and human melanoma cells

**DOI:** 10.3892/mmr.2015.3371

**Published:** 2015-02-18

**Authors:** RAVI PRAKASH SAHU

**Affiliations:** 1 Department of Pathology and Laboratory Medicine, Indiana University School of Medicine, Indianapolis, IN 46202, USA; 2 Department of Dermatology, Indiana University School of Medicine, Indianapolis, IN 46202, USA

**Keywords:** apoptosis, benzyl isothiocyanate, melanoma, platelet-activating factor-receptor, reactive oxygen species

## Abstract

Melanoma cells often express platelet-activating factor receptor (PAF-R), which has been demonstrated to increase metastatic behavior. However, the effect of PAF-R on the responsiveness of melanoma to naturally occurring cytotoxic agents remains to be elucidated. The present study aimed to determine the relative cytotoxicity and mechanism of benzyl isothiocyanate (BITC), a component of cruciferous vegetables, in melanoma cells expressing PAF-R. To evaluate the importance of PAF-R signaling in melanoma cell growth, PAF-R-negative murine B16F10 cells were transduced with a retrovirus containing the cDNA for PAF-R to generate cells stably expressing PAF-R (B16-PAF-R) or an empty vector (MSCV) to generate PAF-R-deficient B16-MSCV control cells. Activation of PAF-R, using the PAF-R agonist, 1-hexadecyl-2-N-methylcarbamoyl-3-glycerophosphocholine, induced an increase in the proliferation of B16-PAF-R cells compared with the B16-MSCV cells. Reverse transcription quantitative polymerase chain reaction revealed the presence of functional PAF-R in human melanoma SK23MEL cells, but not in SK5MEL cells. The present study investigated the effect of BITC treatments on the survival of murine and human melanoma cells, in the presence or absence of functional PAF-R. The results revealed that treatment with BITC decreased the survival rate of the PAF-R-positive and negative murine and human melanoma cells. However, the expression of PAF-R substantially augmented BITC-mediated cytotoxicity in the PAF-R-positive cells at lower concentrations compared with the PAF-R-negative cells. In order to determine the underlying mechanism, flow cytometric analysis was used, which demonstrated a significant increase in the generation of reactive oxygen species (ROS) in the B16-PAF-R cells compared with the B16-MSCV cells, which enhanced apoptosis by BITC, as measured by increased caspase-3/7 luminescence. Notably, the BITC-mediated decreased cell survival rate, increased ROS and increased apoptosis in the B16-PAF-R cells were significantly attenuated by the antioxidant, vitamin C, indicating ROS involvement. Additionally, the WEB2086 PAF-R antagonist, inhibited the BITC-mediated enhancement of apoptosis in the B16-PAF-R cells, indicating a role for PAF-R-signaling in the BITC-mediated effects. These findings indicated that the selectivity of BITC towards PAF-R in melanoma offers a promising chemopreventive agent for PAF-R-positive melanoma treatment.

## Introduction

The incidence of malignant melanoma is rapidly increasing, with the estimated annual mortality rate of >9,000 in the USA (1,2). Melanoma cells often express receptors for multiple growth factors and cytokines, which regulate their growth, including platelet-activating factor-receptor (PAF-R), a G-protein coupled receptor expressed in various types of cell (3–5). The activation of PAF-R has been demonstrated to mediate diverse biological functions in response to various stimuli, including inflammation, immunosuppression and tumorigenesis (6–13).

Previous studies, including ours, have demonstrated that the activation of PAF-R positively modulates melanoma growth, either directly (9–12) or indirectly via host immunomodulation (13). However, in epithelial cell types, activation of PAF-R can increase cell death, induced by pro-oxidative stress reagents (14,15). As melanoma is highly resistant to chemotherapy and radiation therapy (16), identifying novel pathways that can augment cytotoxic agent effects may offer a promising therapeutic approach for melanoma.

Isothiocyanates (ITCs), which are present in cruciferous vegetables, including broccoli and cabbage, possess anti-carcinogenic properties (17,18). Benzyl isothiocyanate (BITC), an analog of ITC suppresses the *in vitro* and *in vivo* growth of various types of cancer (19–22). In melanoma, BITC and other isoforms of ITCs, including allyl and phenyl isothiocyanates and sulforaphane, have been observed to inhibit melanoma cell growth via different mechanisms (23–27). Since many melanomas express functional PAF-Rs and the role of PAF-R in the BITC-mediated suppression of melanoma cells remain to be elucidated, the present study aimed to assess whether the expression of PAF-R can augment the BITC-mediated cytotoxic effects in melanoma cells.

## Materials and methods

### Reagents

A Qiagen RNeasy Mini kit for RNA extraction was purchased from Qiagen Sciences (Germantown, MD, USA), and the Super Script (R) First-Strand Synthesis system for cDNA synthesis was purchased from Invitrogen Life Technologies, Carlsbad, CA, USA). The PAF-R and GAPDH primers and the SYBR Green polymerase chain reaction (PCR) reagents were purchased from SABiosciences (Valencia, CA, USA). A caspase-3/7 activity assay kit was purchased from Promega Corporation (Madison, WI, USA). The WEB2086 PAF-R antagonist, was purchased from Cayman Chemicals Co. (Ann Arbor, MI, USA). All other reagents were purchased from Sigma-Aldrich (St. Louis, MO, USA).

### Cells

Murine B16 cells expressing PAF-R (B16-PAFR), empty vector (B16-MSCV) and human SK23MEL melanoma cells were maintained in RPMI-1640 media (Life Technologies, Grand Island, NY, USA) supplemented with 10% fetal bovine serum (HyClone, GE Healthcare Life Sciences, Logan, UT, USA) and 100 *μ*g/ml mixture of penicillin and streptomycin (Lonza, Walkersville, MD, USA). Human SK5MEL cells were obtained from the American Type Culture Collection (ATCC; Manassas, VA, USA) and cultured in Eagle’s minimum essential medium (ATCC) supplemented with 10% FBS and 100 *μ*g/ml mixture of penicillin and streptomycin.

### Reverse transcription-quantitative PCR (RT-qPCR)

The mRNA expression of PAF-R was analyzed in the human SK5MEL and SK23MEL cells using RT-qPCR and the expression levels were normalized with GAPDH, as described previously (8,13). The B16-PAF-R and B16-MSCV cells were used as positive and negative controls. Briefly, the cells were homogenized using an RLT buffer containing β-mercaptoethanol (Sigma-Aldrich), in a bullet blender (Next Advance, Inc., Averill Park, NY, USA) and carbide beads. The total RNA was extracted using an RNAeasy kit according to the manufacturer’s instructions. The purified RNA was quantified using a Nano Drop 2000 (Thermo Fisher Scientific, Inc., Lafayette, CO, USA) and reverse transcribed with a Super Script cDNA synthesis kit containing random hexamers. The cDNA was analyzed for the PAF-R mRNA using a SYBR green-based, quantitative fluorescent PCR method (6–8,13). The fluorescence was detected using a Step One Real-time PCR machine (Applied Biosystems, Foster City, CA, USA). The quantification of each PCR product was normalized to GAPDH using the 2^−ΔΔCt^ method.

### Cell proliferation

The B16-PAF-R and B16-MSCV cells were plated in 24-well plates (20,000 cells/well) and treated with 1 or 10 nM 1-hexadecyl-2-N-methylcarbamoyl-3-glycerophosphocholine (CPAF) and incubated for 24, 48 or 72 h. The control cells received 0.1% ethanol dissolved in phosphate-buffered saline (PBS;10 *μ*l) only. Following each time point, the cells were trypsinized, washed, resuspended in PBS and stained using 0.4% trypan blue according to the manufacturer’s instructions (Invitrogen Life Technologies). The cell proliferation was assessed using a trypan blue exclusion method and a Countess automated cell counter (Invitrogen Life Technologies).

### Cell survival

The murine or human melanoma cells were seeded into 96-well plates (5,000 cells/well) and treated with various concentrations of BITC, as indicated in respective figures and figure legends. The control cells received 0.1% dimethylsulfoxide (DMSO) treatment. The cell survival rate was assessed 24 h after treatment using a sulforhodamine-B (SRB) assay, as described previously (19). The plates were read at 590nm using a Bio Kinetics plate reader (EL-800; BioTek Instruments, Inc., Winooski, VT, USA).

### Reactive oxygen species (ROS) generation

The B16-MSCV and B16-PAF-R cells (1×10^5^ cells/well) were seeded into 6-well plates for attachment overnight. The cells were treated with H2DCFDA dye (DFC; 5 *μ*M) for 30 min prior to treatment with 2 *μ*M BITC for 0, 5, 10 min or 1 h. In a separate experiment, the cells were pre-treated with vitamin C (5 mM) for 1 h prior to treatment with 2 *μ*M BITC for 10 min. The control cells received 0.1% DMSO treatment. The generation of ROS was analyzed by measuring the number of DCF-positive cells using a flow associated cell sorter and FlowJo software version 9.7.5 (Tree Star, Inc., Ashland, OR, USA).

### Apoptosis

The B16-PAF-R and B16-MSCV cells (1×10^5^ cells/well) were seeded into 6-well plates for attachment overnight. The cells were then either pretreated with 5 mM vitamin C or 10 *μ*M WEB2086 PAF-R antagonist, for 1 h, followed by treatment with 2 *μ*M BITC for 24 h. The control cells received 0.1% DMSO treatment. Apoptosis was quantified using a luminescence caspase-3/7 glo-assay kit and normalized against the total protein, as described previously (28). The protein content of the samples were determined using a Bradford assay kit (Bio-Rad Laboratories, Inc., Hercules, CA, USA).

### Statistical analysis

Each data set is representative of the combined results of at least three independent experiments with similar findings. Data were analyzed using GraphPad Prism software version 5 (GraphPad software, San Diego, CA, USA). Student’s t-test and one-way analysis of variance with a Bonferroni Post-hoc test were used to compare two groups or more than two groups. P<0.05 was considered to indicate a statistically significant difference.

## Results

### Evaluation of the expression of PAF-R and the effect of CPAF the PAF-R agonist on melanoma cell growth

**Figure 1:**
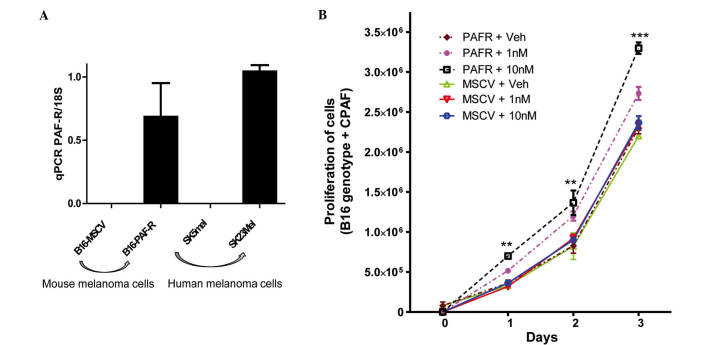
Effect of the PAF-R status on the proliferation of melanoma cells. (A) Evaluation of the presence of PAF-R status using reverse transcription-qPCR in murine and human melanoma cells. (B) B16-PAF-R and B16-MSCV cells were seeded into 24-well plates (20,000 cells/well) and allowed to attach overnight. The cells were treated with either vehicle or 1 or 10nM CPAF PAF-R agonist and cultured for 1, 2 or 3 days. At each time point, the cells were trypsinized and the number of cells were counted using a trypan blue exclusion method. Data are expressed as the mean ± standard deviation of the cell proliferation by CPAF over the number of days. Statistically significant differences were observed between the B16-MSCV and B16-PAF-R cells at 10nM CPAF on days 1, 2 and 3 (^***^P<0.001 and ^**^P<0.01 vs. MSCV + 10 nM group). No significant differences were observed in the baseline growth rate between the B16-MSCV and B16-PAF-R cells. PAF-R, platelet-activating factor-receptor; MSCV, empty vector; qPCR, quantitative polymerase chain reaction; CPAF, 1-hexadecyl-2-N-methylcarbamoyl-3-glycerophosphocholine, VEH, vehicle.

The majority of human melanoma cells express PAF-R, however, the B16F10 murine melanoma cell line, does not (9,13). To determine the role of the PAF-R, a B16-PAF-R cell line was created by transduction of PAF-R-negative B16F10 cells with the MSCV2.1 retrovirus encoding the human leukocyte PAF-R, as described previously (13). B16-MSCV cells were used as a control. The human melanoma cells were then screened for the presence of functional PAF-R. RT-qPCR revealed the presence of functional PAF-R in the human SK23MEL cells (Fig. 1A). By contrast, human melanoma SK5MEL cells deficient in PAF-R were selected and used as a control (Fig. 1A). To evaluate the role of PAF-R in the progression of melanoma, the B16-PAF-R and B16-MSCV cells were treated with the non-metabolizable PAF-R agonist, CPAF, at different doses and time points prior to the assessment of cell proliferation. As shown in Fig. 1B, CPAF-treatment induced the proliferation of B16-PAF-R cells in a dose- and time-dependent manner, compared with the vehicle-treated B16-PAF-R cells. Notably, CPAF had no effect on the proliferation of the B16-MSCV cells at these doses, suggesting that PAF-R was involved in melanoma cell growth.

### Effect of the BITC-mediated cytotoxicity of melanoma cells with regards to the PAF-R

**Figure 2:**
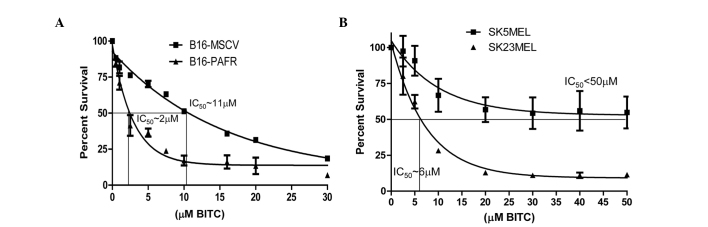
Effect of the expression of PAF-R on the BITC-mediated suppression of murine and human melanoma cell survival. (A and B) B16-PAF-R, B16-MSCV, SK23MEL and SK5MEL cells were treated with either vehicle (0.1% DMSO) or different concentrations of BITC *in vitro* and incubated for 24 h. The cell survival was measured following incubation using an sulforhodamine-B assay. Data are expressed as the mean ± standard deviation and are presented as the percent survival against the BITC treatments. MSCV, empty vector; PAF-R, platelet-activating factor-receptor; BITC, benzyl isothiocyanate; DMSO, dimethylsulfoxide; IC_50_, half maximal inhibitory concentration.

Previous studies have demonstrated that ITC analogs, including BITC, exhibit decreased cytotoxicity towards melanoma cells *in vitro* (half maximal inhibitory concentration 10–20 *μ*M), indicating that higher concentrations are required to achieve efficacy (29). The present study aimed to determine the effect of PAF-R on the BITC-mediated cytotoxicity of melanoma cells. The relative cytotoxicity of BITC in the melanoma cells was compared in the presence or absence of functional PAF-R using murine and human melanoma cells. PAF-R positive, B16-PAF-R and SK23MEL and PAF-R negative, B16-MSCV and SK5MEL cells were treated with different concentrations of BITC for 24 h and cell survival was measured. As shown in Fig. 2A and B, BITC dose-dependently reduced the survival of these cells. However, the expression of the PAF-R augmented the BITC-induced cytotoxicity in the B16-PAF-R (IC_50_ ~2 mM) and SK23MEL (IC_50_ ~6 mM) cells, compared with the PAF-R deficient cells. The IC_50_ of BITC in PAF-R deficient murine B16-MSCV cells was ~11 mM and in the human SK5MEL cells was >50 *μ*M. These findings indicated that PAF-R signaling augments the decreased survival of the melanoma cells, which is elicited by BITC, at a lower concentration than is required for the PAF-R-deficient cells.

### BITC treatment enhances the generation of ROS in PAF-R-expressing melanoma cells

**Figure 3:**
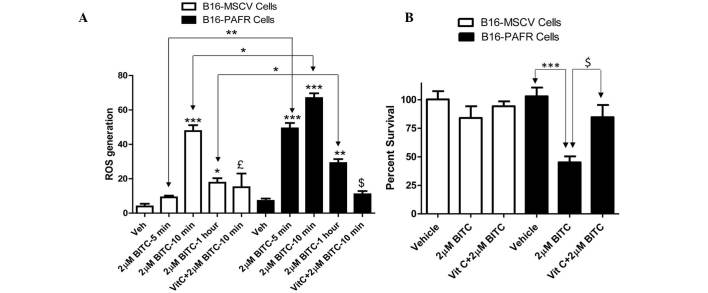
Effect of BITC on the generation of ROS in melanoma cells (A) Effect of BITC treatment (2 *μ*M) on the generation of ROS in the presence and absence of Vit C (5 mM) was analyzed by measuring DCF fluorescence by flow cytometry. Data are represented as the mean ± standard deviation and are presented as the generation of ROS against the different treatment groups. Statistically significant differences were observed between the vehicle and BITC treatment groups and the BITC and vit C + BITC groups (^*^P<0.05; ^£^P<0.01, ^$^P<0.001, ^**^P<0.01 and ^***^P<0.001). (B) The effect of BITC on cell survival rates in the presence and absence of vit C (5 mM) in the B16-PAF-R and B16-MSCV cells was determined using an SRB assay. Data are expressed as the mean ± standard deviation and presented as the percent survival against the different treatment groups. Statistically significant differences were observed between the vehicle and the BITC treated groups and the BITC and vitamin C + BITC treated group (^***^P<0.001; ^$^P<0.001). The control cells received vehicle (DMSO) treatment only. Veh, vehicle; BITC, benzyl isothiocyanate; MSCV, empty vector; PAF-R, platelet-activating factor-receptor; ROS, reactive oxygen species; Vit C, vitamin C.

BITC acts as a pro-oxidative stressor, inducing the generation of ROS as a potent mechanism of tumor cell death (21,22,24,30–32). By contrast, other studies have demonstrated that BITC can also mediate potent antioxidant effects against oxidized low density lipoprotein-induced endothelial dysfunction (33) and inflammation-mediated carcinogenesis (34,35). To determine the mechanism underlying the BITC-induced decreased survival rate of the PAF-R expressing melanoma cells, the effect of BITC on ROS generation was measured. For mechanistic studies, B16-PAF-R and B16-MSCV cells were used as these lines were generated from the same parent (B16F10) cells. As the IC_50_ of BITC in the B16-PAF-R cells was ~2 *μ*M, this concentration of BITC was used to treat the B16-PAF-R and B16-MSCV cells at different time points. The cells were pretreated with the antioxidant, vitamin C (5 mM) for 1 h and subsequently with BITC. As shown in Fig. 3A, BITC treatment induced a significant increase in ROS generation in each of the cell lines. However, in the B16-PAF-R cells, ROS generation occurred as early as 5 min after treatment and was significantly increased compared with the B16-MSCV cells at all time points (Fig. 3A). Treatment with vitamin C inhibited the BITC-induced ROS generation (Fig. 3A) and rescued B16-PAF-R cells (Fig. 3B), indicating a role for ROS in the BITC-induced suppression of the B16-PAF-R cells.

### PAF-R augments BITC-induced apoptosis in the B16-PAF-R cells

**Figure 4:**
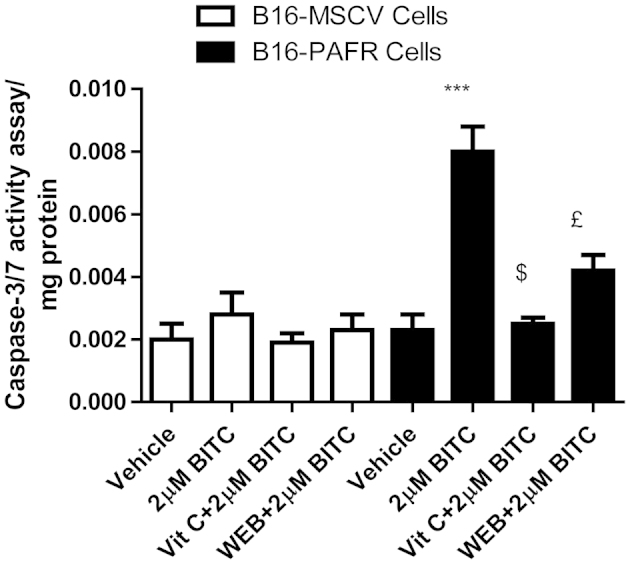
Effect of PAF-R on BITC-mediated apoptosis in the melanoma cells. The B16-MSCV and B16-PAF-R cells were pre-treated either with Vit C (5 mM) or the WEB2086 PAF-R antagonist (10 *μ*M), for 1 h followed by treatment with 2 *μ*M BITC for 24 h. Apoptosis was assessed using a fluorogenic caspase-3/7 activity assay kit. Data are expressed as the mean ± standard deviation and are presented as caspase-3/7 activity/mg protein against the different treatment groups. Statistically significant differences were observed between the vehicle and BITC, BITC and Vitamin C + BITC and BITC and WEB + BITC (^***^P<0.001 vs. vehicle group; ^$^P<0.001 vs. 2 *μ*M BITC group and ^£^P<0.01 vs. 2 *μ*M BITC group). PAF-R, platelet-activating factor-receptor; BITC, benzyl isothiocyanate; MSCV, empty-vector; Vit C, vitamin C; WEB, WEB2086.

The induction of apoptosis in malignant cells has been revealed as a major mechanism of chemopreventive/therapeutic drug-mediated cell death (12,15,19,30–33). To determine whether the expression of PAF-R augments the BITC-mediated decrease in B16-PAF-R cell survival via ROS generation, apoptosis induction was measured. The B16-PAF-R and B16-MSCV cells were treated with 2 *μ*M BITC for 24 h in the presence or absence of either vitamin C or the WEB2086 PAF-R antagonist. As shown in Fig. 4, BITC-treatment resulted in a significant apoptotic response in the B16-PAF-R cells compared with the B16-MSCV cells. The induction of apoptosis in the B16-PAF-R cells was significantly attenuated following treatment with vitamin C and WEB2086. These results indicated the involvement of PAF-R signaling in the BITC-induced decrease in growth of the B16-PAF-R cells mediated via increased ROS generation and induction of apoptosis.

## Discussion

The activation of PAF-R is important in diverse biological processes, including regulating the growth of melanoma (3–13), and the majority of types of melanoma express PAF-R (9) and are resistant to the currently used chemotherapeutic agents (16). Therefore, the present study investigated the efficacy of BITC against melanoma cells, in those either expressing PAF-R or not. Human melanoma cells, either deficient in or expressing PAF-R, and murine melanoma B16F10 cells, which have been extensively used in chemotherapy studies and lack functional PAF-R expression, were genetically modified to produce cells stably expressing PAF-R or vector-controls as suitable model systems.

Treatment with CPAF increased the proliferation of the PAF-R-expressing B16-PAF-R cells in a dose- and time-dependent manner compared with the B16-MSCV cells, confirming that PAF-R activation enhances the growth of melanoma cells. By contrast, BITC-treatment reduced the survival rate of the murine and human melanoma cells in a dose-dependent manner. However, the presence of functional PAF-R potentiated the cytotoxicity of BITC in the B16-PAF-R murine and human SK23MEL melanoma cells, requiring relatively lower doses than were required for the PAF-R deficient cells. This indicated that the expression of PAF-R results in enhanced cytotoxicity of the melanoma cells by BITC.

Generation of ROS is an early event following chemotherapy, that is important in inducing apoptosis in malignant cells (15,30–32). In this context, treatment with BITC at doses that substantially reduced the survival of the B16-PAF-R cells, increased the generation of ROS and the levels of apoptosis compared with the B16-MSCV cells. These effects were attenuated by vitamin C, suggesting the involvement of ROS in the BITC-induced decreased survival of B16-PAF-R cells. These data are consistent with previous studies, which demonstrated that the BITC-induced suppression of malignant cells was mediated via ROS generation and inhibited by free radical quenchers (30). The present study demonstrated that BITC-treatment resulted in an increased level of apoptosis of the B16-PAF-R cells via ROS and confirmed the role of the PAF-R activation. The BITC-induced increase in apoptosis in the B16-PAF-R cells was inhibited by the WEB2086 PAF-R antagonist, confirming the involvement of PAF-R signaling in this process. The ability of PAF-R signaling to promote the proliferation of the B16-PAF-R cells and to augment BITC-mediated apoptosis was in agreement with previous studies, which observed that CPAF-treatment induced increased proliferation in a PAF-R-negative KB epithelial cell line, which was genetically modified to stably express the functional PAF-R (KBP), however this did not occur in retroviral vector-transduced control KBM cells (36). Similarly, the expression of PAF-R augmented ultraviolet B (UVB)-mediated apoptosis of PAF-R-positive KBP but not PAF-R-negative KBM cells (14). This increased susceptibility of the KBP cells towards UVB-mediated enhanced apoptosis was inhibited by antioxidants and PAF-R antagonists. Additionally, PAF-R activation augmented chemotherapy-induced cytotoxicity in human carcinoma cell lines (15). These previous studies confirm the involvement of the PAF-R signaling in mediating pro-oxidative stressors including BITC-mediated increase in melanoma cell apoptosis.

In conclusion, naturally occurring ITCs have been demonstrated to possess anticarcinogenic properties, however, the lack of specific oncogenic targets and the use of higher concentrations to achieve optimum therapeutic efficacy have made them unsuitable. Therefore, the selection of potent analogs, which can target specific signaling pathways may offer more effective agents against malignant cells. The present study demonstrated that BITC suppressed the growth of melanoma cells and that this was augmented by the activation of PAF-R through the ROS-mediated pathway. Collectively, these data suggest that BITC may be used as a novel chemotherapeutic agent against PAF-R-expressing melanoma cells.

## Abbreviations

PAF: platelet-activating factor
PAF-R: PAF-receptor
CPAF: 1-hexadecyl-2-N-methylcarbamoyl-3-glycerophosphocholine
BITC: benzyl isothiocyanate
ROS: reactive oxygen species
FACS: fluorescence-activated cell sorting
mRNA: messenger RNA
PBS: phosphate-buffered saline
RT-qPCR: reverse transcription-quantitative polymerase chain reaction
